# Chromogenic Assay Is More Efficient in Identifying α-Amylase Inhibitory Properties of Anthocyanin-Rich Samples When Compared to the 3,5-Dinitrosalicylic Acid (DNS) Assay

**DOI:** 10.3390/molecules28176399

**Published:** 2023-09-01

**Authors:** Sadia Zulfiqar, Federica Blando, Caroline Orfila, Lisa J. Marshall, Christine Boesch

**Affiliations:** 1 School of Food Science and Nutrition, Faculty of Environment, University of Leeds, Leeds LS2 9JT, UK; sadiazulfiqar777@yahoo.com (S.Z.); c.orfila@leeds.ac.uk (C.O.); l.j.marshall@leeds.ac.uk (L.J.M.); 2 Institute of Sciences of Food Production (ISPA), National Research Council (CNR), Via Prov.le Lecce-Monteroni, 73100 Lecce, Italy; federica.blando@ispa.cnr.it

**Keywords:** α-amylase, anthocyanins, direct chromogenic assay, DNS assay, carbohydrate digestion

## Abstract

The inhibition of carbohydrate digestion by plant bioactive compounds is a potential dietary strategy to counteract type 2 diabetes. Indeed, inhibition of α-amylase, a key enzyme that carries out the bulk of starch digestion, has been demonstrated for a range of bioactive compounds including anthocyanins; however, sample pigmentation often interferes with measurements, affecting colorimetric assay outcomes. Therefore, the present study compared the performance of a direct chromogenic assay, using 2-chloro-4 nitrophenyl α-D-maltotrioside (CNPG3) as a substrate, with the commonly used 3,5-dinitrosalicylic acid (DNS) assay. The direct chromogenic assay demonstrated a 5–10-fold higher sensitivity to determine α-amylase inhibition in various samples, including acarbose as a reference, pure anthocyanins, and anthocyanin-rich samples. The IC_50_ values of acarbose presented as 37.6 μg/mL and 3.72 μg/mL for the DNS assay and the direct chromogenic assay, respectively, whereas purified anthocyanins from blackcurrant showed IC_50_ values of 227.4 µg/mL and 35.0 µg/mL. The direct chromogenic assay is easy to perform, fast, reproducible, and suitable for high-throughput screening of pigmented α-amylase inhibitors.

## 1. Introduction

Anthocyanins, a sub-class of flavonoids, are water-soluble naturally occurring bioactive compounds responsible for the red to dark blue colour of most flowers, fruits, and vegetables [1,2]. Over the last few decades, anthocyanins have been extensively studied for their diverse beneficial health effects such as antioxidant, antibacterial, antiviral, anti-inflammatory, and antidiabetic activity [3]. Epidemiological studies have suggested that the consumption of foods rich in anthocyanins lowers the risk of diabetes and diabetic complications [4]. Type 2 diabetes mellitus is a chronic metabolic disorder characterized by hyperglycaemia resulting from pancreatic β-cell dysfunction or impaired insulin secretion, with or without insulin resistance. The prevalence of diabetes is increasing worldwide, with estimates of 10.5% (536.6 million people) in 20–79-year-olds in 2021, rising to 12.2% (783.2 million) in 2045 [5]. The intake of diets and foods rich in bioaccessible starches and sugars are positively associated with elevated postprandial blood glucose concentrations [6].

Dietary carbohydrates are hydrolysed by salivary and pancreatic α-amylases in the mouth and small intestine, respectively, to smaller fragments such as maltose and maltodextrins. The resultant products of α-amylase action are further hydrolysed by intestinal brush border α-glucosidases into absorbable glucose units [7]. Although different amylolytic enzymes participate in the process of starch breakdown, the contribution of α-amylase is the most important for the initiation of this process [8]. Therefore, the inhibition of starch hydrolysis through α-amylase inhibition seems to be an effective way to slow down and control glucose availability, hence modulate postprandial hyperglycaemia.

Antidiabetic drugs, such as acarbose, miglitol, and voglibose, have been approved for their use in diabetes management. They reduce postprandial hyperglycaemia by inhibiting the activity of digestive enzymes [9,10]. However, these inhibitors demonstrate side effects such as nausea, abdominal pain, and flatulence, emphasizing the need for alternative approaches. Indeed, in recent years, polyphenols and in particular anthocyanins have been heavily investigated as potential α-amylase (and α-glucosidase) inhibitors for the management and risk reduction of type 2 diabetes as alternatives to pharmaceutical treatments such as acarbose [6,11,12,13,14,15].

There are several methods reported in the literature to determine the α-amylase inhibitory properties of polyphenolic compounds. These include methods to detect reducing sugars, such as the 3,5-dinitrosalicylic acid (DNS) assay and the Nelson–Somogyi reagent, as well as the iodine–starch test, turbidity test, and chromogenic methods [16]. Chromogenic methods use chemically modified derivatives of polymers and malto-oligosaccharides of varying chain length linked to chromophores, such as 4-nitrophenyl or 2-chloro-4-nitrophenyl [16,17,18,19,20]. One of the most commonly used assays for measuring α-amylase inhibition involves the DNS reagent for the detection of reducing sugars. The carbonyl end of the reducing sugars participates in an oxidation–reduction reaction with the aromatic DNS reagent to yield the deep-orange-coloured 3-amino-5-nitrosalicylic acid (ANS), which absorbs light strongly at 540 nm [21]. However, the reducing potential of dietary polyphenols enables them to take part in the oxidation–reduction reaction, interfering with the colour development and thereby potentially impacting on the assay results. Solid-phase extraction has been recommended to minimize the colour interference caused by polyphenols [20]. The molecular size of the substrates (starch, amylose, amylopectin) is another factor affecting the outcome of the DNS assay which results in the under- or overestimation of reducing power [16]. Additionally, the structural complexity among many of the natural starches makes them less suitable for detailed kinetic and inhibition studies. In order to address these problems, and to facilitate the detection of enzymatic activity, many smaller, defined substrates have been developed [22].

There is a rationale to explore the use of alternative assays using synthetic substrates in order to determine the inhibition of α-amylase activity by pigmented polyphenols, i.e., anthocyanins. Recent research [19] highlighted the importance of a High-Performance Anion-Exchange Chromatography coupled with Pulsed Amperometric Detector (HPAE-PAD) technique using a small-chain substrate (maltoheptaoside) as a more precise and accurate method for accessing the α-amylase inhibitory potential of polyphenols with minimum interference. However, the preparation of different standards and the use of expensive instruments are limitations of this method [19]. Some studies have reported the use of short-chain chromogenic substrates such as 2-chloro-4 nitrophenyl α-D-maltotrioside (CNPG3) to measure α-amylase inhibitory properties of bioactive compounds [16,17,23]. Research on α-amylase substrate length demonstrated that α-amylase effectively hydrolyses maltotrioside to maltose and glucose at concentrations lower than 100 mM [24], supporting the choice of the substrate length. Whilst chromogenic substrates have been available for a number of years, they were mainly utilized for the measurement of amylase activity in blood and urine [25]; hence, there is a lack of information on the performance of this assay to determine enzyme inhibitory properties of bioactive compounds such as polyphenols and other pigments.

On the basis of the above-mentioned shortcomings in measuring pigmented polyphenol inhibitory activity, the aim of the current study was to test the performance of a direct chromogenic assay that utilizes CNPG3 as the substrate, in comparison with the DNS assay, to determine the α-amylase inhibitory properties of anthocyanin-rich extracts and purified anthocyanins.

## 2. Results and Discussion

### 2.1. Comparison of DNS Assay and Direct Chromogenic Assay for α-Amylase Inhibition by Acarbose

**Figure 1:**
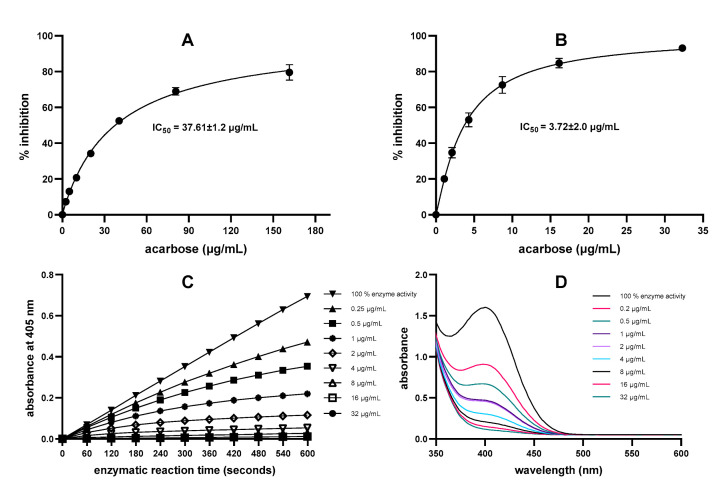
Dose-dependent effect of acarbose on inhibition of α-amylase determined by (**A**) DNS assay using amylose as substrate and (**B**) by direct chromogenic assay using CNPG3 as a substrate. The kinetic data of the measurement (**C**) and wavelength scan (**D**) demonstrate the linear relationship of absorbance with substrate cleavage/product formation at 405 nm over time showing *p*-nitrophenol product formation at different acarbose concentrations.

Acarbose, a well-known α-amylase and α-glucosidase inhibitor, is a commonly used antidiabetic drug (Glucobay, Precose) and is typically used as a positive control when assessing the enzyme inhibitory properties of plant extracts in vitro [15,26]. Indeed, in the present study, acarbose was used as a reference compound in both assays, allowing the comparison of enzyme inhibition using the DNS assay and the direct chromogenic method. While acarbose inhibited α-amylase activity in both assays, the results from the DNS assay (covering an acarbose concentration range from 1 to 100 µM) demonstrated 50% enzyme inhibition (IC_50_) at 37.6 μg/mL (Figure 1A). These results are in line with the other literature reporting IC_50_ values of 11.7 µg/mL [15] and 77.5 µg/mL [27] for α-amylase inhibitory potential of acarbose using the DNS assay. In contrast to the DNS data, the results from the direct chromogenic assay showed a much lower acarbose IC_50_ of 3.72 μg/mL (Figure 1B). This outcome is in agreement with previous research demonstrating acarbose inhibition of PPA under similar assay conditions using a chromogenic assay [8,17,23,26]. Recently, Xie et al. [28] reported the inhibition of human pancreatic amylase by acarbose (IC_50_ 0.47 µg/mL) using a similar substrate.

The increase in absorbance from the release of *p*-nitrophenol was recorded at 1 min intervals over the 10 min reaction period and plotted against time. For acarbose (Figure 1C), the results showed linearity with r > 0.99 for all samples. In addition, wavelength scans (350–600 nm) confirmed the dose-dependent reduction in reaction product formation in the presence of the inhibitor acarbose, whereas maximum *p*-nitrophenol was released without acarbose, as shown in Figure 1D.

### 2.2. Comparison of DNS Assay and Direct Chromogenic Assay for α-Amylase Inhibition by Anthocyanin-Containing Samples

**Figure 2:**
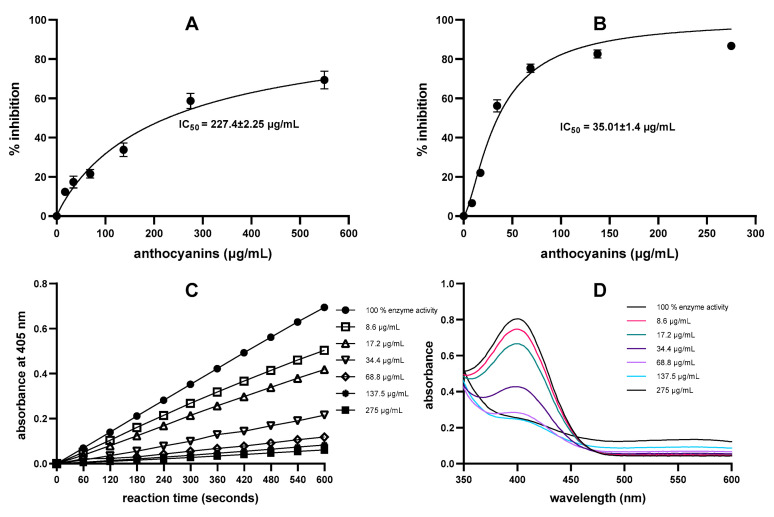
Dose-dependent α-amylase inhibition by blackcurrant determined by (**A**) DNS assay (0–1000 μg/mL) and (**B**) direct chromogenic assay (0–500 μg/mL). Substrate cleavage and product formation were monitored through kinetic measurement recording (**C**) and wavelength scan (**D**).

In this study, nine anthocyanin-rich extracts/concentrates and two purified anthocyanins (cyanidin and cyanidin-3-O-galactospyranoside) were tested for α-amylase inhibitory activity using both assays. The blackcurrant sample, which consisted of anthocyanins only (>95% purity) [29], inhibited enzyme activity in both assays, with IC_50_ 227 µg anthocyanins/mL and IC_50_ 35 µg/mL for the DNS assay and direct chromogenic assay, respectively (Figure 2A,B). Similar to acarbose, the kinetic measurement (Figure 2C) and wavelength scan (Figure 2D) of blackcurrant samples demonstrated a linear response (r > 0.99) and identity of *p*-nitrophenol release in the presence of different concentrations of blackcurrant, indicating that the pigmentation of the anthocyanins did not interfere with the measurement at 405 nm.

Anthocyanin-rich concentrates from pomegranate and blueberry showed strong α-amylase inhibition when assessed with the direct chromogenic assay, whereas weak and no inhibition was found for cherry and hibiscus, respectively (Table 1). In contrast, the DNS assay data did not support inhibitory properties for any of these extracts, even when SPE was employed to remove potentially interfering polyphenols prior to the DNS reagent addition, which was recommended previously [20]. The intake of anthocyanin-rich extracts or foods such as blackcurrant, cranberry juice, and mixed berries has been shown to reduce postprandial glycaemia and insulinemia [30,31,32]. The most likely and quantitatively most relevant mechanism by which fruit extracts or concentrates reduce postprandial hyperglycaemia is the attenuation of starch breakdown through α-amylase and α-glucosidase inhibition [15,33,34]. It has been reported that anthocyanins such as cyanidin-3-glucoside, cyanidin-3,5-glucoside, cyanidin-3-rutinoside, and peonidin-3-glucoside exert in vitro inhibition towards α-amylase [35]. For example, cyanidin-3-rutinoside, a major anthocyanin found in many fruits such as sweet cherry and blackcurrant, is a strong α-amylase inhibitor (IC_50_ 24.4 μM) [15].

Enzyme inhibitory properties are known for a number of polyphenols. In fact, the presence of other polyphenols in the anthocyanin-rich extracts may have contributed to inhibitory activities. As indicated in Table 2, there are marked differences in the total polyphenol and anthocyanin concentration of the different samples. Pomegranate has the highest amount of total polyphenols, and this may have contributed to its lower IC_50_ value. However, whilst inhibitory effects towards α-amylase have been related to anthocyanins, they may also be due to other compounds. Indeed, in the present study, α-amylase-inhibitory properties were found to be independent of anthocyanin content, i.e., pomegranate, which has a low anthocyanin content (Table 2) but has demonstrated the strongest inhibitory properties among the berry samples. Therefore, the presence of other compounds in fruit concentrates might be responsible for the differing inhibitory potential against α-amylase, which, in the case of pomegranate, might be due to ellagitannins, especially punicalin and punicalagin [41]. Similar findings were also reported for strawberry and raspberry extracts [42]. However, Grussu et al. [43] has emphasized that ellagitannins might not be solely responsible for amylase inhibition. It was observed that both yellow raspberries and red raspberry extracts inhibited α-amylase to the same extent, questioning further the contribution of anthocyanins. The results of the present study using blackcurrant-derived anthocyanins, however, confirm the potential of anthocyanins to inhibit the α-amylase enzyme.

Furthermore, it has been reported that the efficiency of the DNS assay may be compromised by the presence of intrinsic sugars (glucose, fructose) [44] in fruit extracts, which may react with the DNS reagent and generate higher background absorbance. Indeed, the sugar content in all berry concentrates was >500 mg/mL, with mainly glucose and fructose, but very low in hibiscus (Table 2). Whilst the current measurements considered the individual background of the different samples through separate controls (sample lacking enzyme and substrate), it cannot be entirely ruled out that an intrinsic sugar content may have impacted the results. In contrast, the chromogenic assay directly records the continuous absorbance increase at the target wavelength for *p*-nitrophenol (405 nm) for individual samples. It is therefore not expected that this assay would be impacted by the presence of pigments and sugars in the samples.

Crude extracts from mahaleb cherry (*Prunus mahaleb*) and black carrot (*Daucus carota* var. *atrorubens*) and corresponding purified anthocyanin fractions were also analysed in this study. Mahaleb cherry contains simple cyanidin glycosides in contrast to black carrot consisting mainly of acylated cyanidin glycosides. These samples have been reported for their antioxidant, anti-inflammatory, and cardiovascular protective properties [40]. However, as shown in Table 1, the mahaleb cherry extract was a weak inhibitor at the indicated concentrations, and no inhibitory effect was evident for black carrot.

Cyanidin and its glycosides are one of the most naturally occurring anthocyanins. In the present study, cyanidin and a glycoside were tested in both assays. Whilst the DNS assay indicated only slight inhibition (7%) at concentrations up to 435 μM for cyanidin, an IC_50_ of 141 μM could be established using the chromogenic assay. In contrast, cyanidin-3-O-galactopyranoside showed a three times higher IC_50_ value with the same assay (414 μM). These findings are in alignment with the literature demonstrating that increasing numbers of sugar moieties attached to the anthocyanin molecule reduce inhibitory activity. For example, cyanidin and cyanidin-3-glucoside demonstrated higher inhibitory activity compared to cyanidin-3,5-diglucoside (IC_50_ 109.2 µg/mL, 145.5 µg/mL, and >600 µg/m/L, respectively, using the DNS assay) [27]. The current study also investigated, apart from cyanidin and cyanidin-3-galactopyranoside, the contribution of anthocyanin metabolites to amylase inhibition, i.e., gallic acid, chlorogenic acid, and protocatechuic acid. Even at high concentrations of 1000 μg/mL, we were unable to determine inhibition of α-amylase for any of these compounds,), which is in line with a recent report on absent α-amylase inhibitory properties of chlorogenic and phenolic acids at relevant concentrations [45].

Apart from glycosylation, other differences in anthocyanin structure may impact α-amylase inhibition properties [28]. For example, increased inhibitory activities were observed for anthocyanins with more hydroxyl groups [18,23]. Indeed, it has been shown that delphinidin (three hydroxyl groups on the B ring) exerted more efficient enzyme inhibition compared to cyanidin and petunidin, which have two hydroxyl moieties [46]. In addition, a significantly improved inhibition of human salivary amylase was evident for cyanidin-3-glucoside (IC_50_ 180 ± 20 µM) compared to malvidin-3-glucoside (IC_50_ 675 ± 73 µM). Similarly, Sui, Zhang, and Zhou [35] reported a lower IC_50_ (24 ± 3 µM) for cyanidin-3-glucoside as compared to peonidin-3-glucoside (IC_50_ 75 ± 7 µM). As well, it has been reported that acetylated anthocyanins in comparison to non-acylated representatives are better inhibitors for α-amylase as well as α-glucosidases [47,48].

The results of the current study demonstrated the increased sensitivity (5–10 fold) of α-amylase inhibitory properties of acarbose as well as some of the anthocyanins and anthocyanin-rich extracts when the direct chromogenic assay was used in comparison to the DNS assay. The reason for the discrepancy is likely related to differing substrate properties [49]. For example, when using amylose and amylopectin as substrate, acarbose showed IC_50_ values of 3.5 μM and 10 μM [20], respectively, under the same assay conditions, emphasizing the link between increased substrate complexity and enzyme activity. Indeed, our preliminary data revealed, when using potato starch as a substrate instead of amylose, the IC_50_ values of acarbose increased from 58.3 to 254 μM. In contrast, CNPG3 consists of a tri-glucoside linked to a chromogen, a short-chain and linear molecule that may be well accessible for α-amylase but actually may have less affinity to the enzyme compared to a longer substrate [19].

### 2.3. Mode of Inhibition of Anthocyanins on α-Amylase Activity

**Figure 3:**
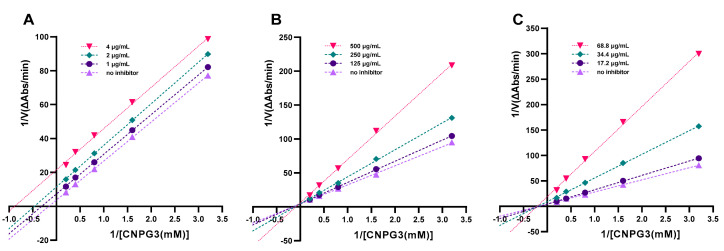
Lineweaver–Burk plots demonstrating inhibition type of (**A**) acarbose, (**B**) cyanidin-3-O-galactoglucoside, and (**C**) blackcurrant against pancreatic α-amylase.

While IC_50_ values showed the potency of natural compounds towards enzyme inhibition, further valuable information was obtained by determining the kinetics of inhibition by natural extracts or isolated compounds. Studies on the kinetics of inhibition are major tools that enable us to distinguish between different inhibitory mechanisms. The initial velocity ‘V’ of the hydrolysis reactions catalysed by pancreatic α-amylase was measured at various substrate concentrations [S] (0–5 mM) in the presence or absence of inhibitors. The Lineweaver–Burk plots, shown in Figure 3, demonstrate significant changes in Km and Vmax under these conditions. In the presence of acarbose (1, 2, 4 μg/mL), both the Vmax and Km decreased, indicating uncompetitive enzyme inhibition (Figure 3A). With cyanidin-3-O-galactopyranoside in the reaction mixture (125, 250, and 500 μg/mL), Vmax values remained constant, whereas Km values increased to 7.95, 10.79, and 23.46 mM, respectively (Figure 3B), indicating that cyanidin-3-O-galactopyranoside is a competitive inhibitor. The results are consistent with a previous study demonstrating that purified anthocyanins competitively inhibited the hydrolysis of a synthetic substrate (CNPG3) by human salivary amylase [50]. Similarly, another study found that anthocyanins such as cyanidin-3-glucoside, cyanidin-3,5-glucoside, cyanidin-3-rutinoside, and peonidin-3-glucoside inhibited the activity of PPA competitively, measured via the DNS method [35]. In competitive inhibition, the inhibitor and the substrate compete for the active site of the enzyme, suggesting that, in the current study, anthocyanins might compete with CNPG3 and exert their inhibitory effect via binding with the active site of α-amylase.

In contrast, with blackcurrant anthocyanins, the Km remained unaffected, whereas Vmax decreased, showing mixed-type inhibition of α-amylase (Figure 3C). Previously, mixed-type inhibition (competitive and non-competitive) was observed for anthocyanin-rich bilberry extract against PPA, measured using the DNS method, suggesting that inhibitors interact with the active site of the enzyme, but they are also bound with enzyme–substrate complexes to create ternary inhibitor–enzyme–substrate complexes, resulting in decreased enzyme activity [51]. Indeed, pre-incubation of the substrate or the enzyme with the inhibitor may affect enzyme activity through the formation of substrate–inhibitor or enzyme–inhibitor complexes [52].

The Km value (Michaelis constant) indicates the affinity of an enzyme for a substrate. The lower the Km value, the higher the affinity of the enzyme for the substrate. Compared to native starch, short-chain *p*-nitrophenyl-linked maltose derivatives such as CNPG3 exhibit a lower affinity for amylase [16,19,53]. The Km value (4.4 mg/mL) for CNPG3, as determined in the present study, was indeed higher than that for maize starch (0.73 mg/mL) [51], confirming the lower affinity of CNPG3 for α-amylase. Similarly, Nyambe-Silavwe, Villa-Rodriguez, Ifie, Holmes, Aydin, Jensen, and Williamson [20] reported a lower Km value (6.68 mg/mL) for amylose compared to amylopectin (12 mg/mL) at substrate concentrations of 1 mg/mL. These findings highlight the importance of clearly reporting the substrate and assay conditions when interpreting IC_50_ and enzyme kinetic parameters.

## 3. Materials and Methods

### 3.1. Chemicals and Reagents

3,5-Dinitrosalicylic acid, potassium sodium tartrate, sodium hydrogen phosphate, porcine pancreatic α-amylase (PPA), amylose (from potato starch), and 2-chloro-4 nitrophenyl α-D-maltotrioside (CNPG3), as well as quercetin, gallic acid, chlorogenic acid, protocatechuic acid, and cyanidin, were all purchased from Sigma-Aldrich Co., Ltd., Dorset, UK. Oasis MAX cartridges 1 mL (30 mg) were purchased from Waters Corporation Ltd., Milford, MA, USA. Acarbose was purchased from Acros Organics (Fisher Scientific Ltd., Loughborough, UK). Blueberry, cherry, and pomegranate (100% fruit concentrate) were kindly provided by Active Edge Nutrition Ltd., Hounslow, UK, and hibiscus concentrate was kindly donated by IBIS Organics, Carlisle, UK. Blackcurrant (purified anthocyanin extract, >95% purity) was kindly provided by Keracol, University of Leeds [29]. Cyanidin-3-O-galactospyranoside was kindly donated by Biolink Group, Sandnes, Norway.

Crude and purified anthocyanin-containing samples of mahaleb cherry (*Prunus mahaleb* L.) and black carrot (*Daucus carota* L. ssp. *sativus* var. *atrorubens* Alef.) were prepared at ISPA-CNR, Italy, as previously described [40].

### 3.2. Preparation and Analysis of Samples

Concentrated stock solutions of pure anthocyanins and purified/non-purified anthocyanin-rich extract samples were prepared in acidified methanol (0.1% trifluoracetic acid) and stored in aliquots at −20 °C. The working dilutions of compounds and extracts for enzyme assays were prepared in 20 mM phosphate-buffered saline (PBS), pH 6.9. Blueberry, cherry, pomegranate, and hibiscus concentrates were diluted 1:10 with water and centrifuged (4000× *g*; 4 °C) for 5 min. The supernatants were further diluted in PBS buffer for enzyme assays. The amylase inhibitor acarbose was employed as a positive control, prepared as stock solution in DMSO (10 mM) and further diluted in buffer, in an assay-dependant range. The total polyphenol and anthocyanin content in fruit concentrates, as well as the sugar content, were determined according to previous protocols [54,55,56].

### 3.3. DNS Assay

The inhibition of α-amylase activity was determined using the DNS assay according to previously described procedures [20,56]. In brief, a 100 µL sample (extract, pure compound or acarbose), diluted in PBS, was incubated with 200 µL of amylose solution (2.5 mg/mL) for 10 min at 37 °C. Subsequently, 200 µL PPA (1.25 U/mL) was added to start the reaction and incubated at 37 °C for 10 min. The samples were then placed in a water bath (100 °C, 10 min) to stop the enzyme reaction, subsequently transferred to ice, cooled down, and centrifuged at 4000× *g* for 5 min.

In order to remove potentially interfering compounds such as anthocyanins and other polyphenols that might react with the DNS reagent, solid phase reaction (SPE) was applied [20]. Briefly, Oasis MAX cartridges (30 mg) were preconditioned (1 mL water, 1 mL methanol) and then dried under vacuum for 10 min. The samples that underwent the enzymatic reaction above were applied to the cartridge, and the flow through was collected. Then, 1 mL DNS reagent was added to each SPE-purified flow through sample (500 μL), and samples were heated for 10 min at 100 °C to aid the reaction. Subsequently, samples were cooled down to room temperature, diluted to an appropriate absorbance range, transferred to wells in 96-well plates (2 × 250 µL per sample), and absorbance was recorded at 540 nm using Tecan Spark 10 M multimode microplate reader (TECAN, Mannedorf, Switzerland). Enzyme inhibition was calculated as a percentage of the 100% activity control (absence of inhibitor) and considering individual sample background (absence of enzyme and substrate).

### 3.4. Direct Chromogenic Assay

The inhibition of α-amylase activity was measured according to the method described by Kalita, Holm, LaBarbera, Petrash, and Jayanty [26] with some modifications. Briefly, in wells of a 96-well plate, 2 × 50 µL of each sample (extracts, pure compounds, acarbose) were incubated with 100 µL of PPA solution (1 U/mL) in 20 mM phosphate buffer (pH 6.9) for 10 min at 37 °C. This was followed by the addition of 50 µL of 2 mM CNPG3 substrate, diluted in phosphate buffer. The change in absorbance, indicating the release of *p*-nitrophenol as a reaction product, was recorded in 1 min intervals at 405 nm over a 10 min period (at 37 °C) using a Tecan Spark 10 M microplate reader. Reaction product scans (360–600 nm) were performed at the end of each experiment. Enzyme activity and inhibition (%) were calculated from the absorbance difference of each sample, in reference to the non-inhibited control.

In preliminary experiments, the optimum enzyme and substrate concentrations for the assay were determined using different concentrations of α-amylase (0.25, 0.5, and 1 U/mL) and CNPG3 (0, 0.625, 1.25, 2.5, and 5 mM). The linearity of enzyme reaction was monitored over 20 min under optimized enzyme and substrate concentrations, and incubation time for enzymatic reaction was defined. In order to determine Km and Vmax, the selected enzyme concentration (1 U/mL) was incubated with varying concentrations of substrate (0–5 mM). The rate of enzymatic reaction (V) was calculated by dividing the change in absorbance (at 405 nm) over time. The Lineweaver–Burk double reciprocal plot was obtained by plotting the 1/V against reciprocal of substrate (1/[S]), and Km and Vmax were calculated.

To detail the inhibitory mechanism, a series of experiments were conducted by maintaining PPA at 1 U/mL and varying the concentration of inhibitors (acarbose, blackcurrant, and cyanidin-3-O-galactopyranoside) and substrate CNPG3 (0–5 mM). A Lineweaver–Burk plot was constructed for every inhibitor, and Km and Vmax were calculated.

### 3.5. Statistical Analysis

All experiments were conducted at least in triplicates and analysed using Prism software program v. 9 (GraphPad Software, San Diego, CA, USA). The IC_50_ values (concentration at 50% enzyme inhibition) of pure compounds and purified and non-purified extracts were calculated using a non-linear regression model. The calculated inhibition rates at every concentration of anthocyanins/anthocyanin-rich extracts and acarbose were fitted with a nonlinear curve fit in GraphPad Prism. The IC_50_ values were then obtained from the respective curve. The mode of enzyme inhibition was graphically determined using the Lineweaver–Burk plot through nonlinear regression of all data sets at once using GraphPad Prism software.

## 4. Conclusions

This study tested the performance of a direct chromogenic assay that utilizes CNPG3 as the substrate, in comparison with the DNS assay, to determine the α-amylase inhibitory properties of anthocyanin-rich extracts and purified anthocyanins. The chromogenic assay consistently showed lower IC_50_ compared to the DNS assay. Due to this higher sensitivity, as well as a lack of interference from pigmented and sugar compounds, this method should be favoured for enzyme inhibition measurements in pigmented samples. The microplate format makes the assay efficient for high-throughput screening purposes, with application in academic and industrial research to screen for potential antidiabetic components and assess food formulations for functional properties. However, it should be noted that α-amylase has lower affinity for CNPG3 compared to natural starch substrates. The inhibitory activities of the various anthocyanin samples varied from sample to sample, with the highest potential for blackcurrant and blueberry. Variations in outcomes linked to anthocyanins may be associated with anthocyanin structure, in particular hydroxylation, glycosylation, and acylation pattern. Enzyme inhibitory properties may also be affected by the presence of other inhibitors in the extracts, such as other polyphenols. Further investigations elucidating the inhibitory potential of differently structured anthocyanins on α-amylase, their mode of inhibition, and validation against in vivo inhibition will need to be conducted for the most effective use of anthocyanins in the prevention and management of diabetes.

## Figures and Tables

**Table 1:** Comparison of α-amylase inhibitory properties of anthocyanin-rich extracts and individual anthocyanins using direct chromogenic assay versus DNS assay.

Extracts (Pure/Crude)	DNS Assay (IC_50_/% Inhibition)	Direct Chromogenic Assay (IC_50_/% Inhibition)	Predominant Anthocyanins
Blackcurrant (purified) *	227.4 ± 2.25 µg/mL	35.01 ± 1.4 µg/mL	Dp3rut, Cy3rut, Dp3glu, Cy3glu [29]
Blueberry	NO (645 μg/mL)	80.44 ± 2.0 µg/mL	Dp3glu, Mv3glu, Dp3glc, Mv3glc [36]
Cherry	NO (at 536 μg/mL)	30% at 268 μg/mL	Cy3rut, Cy3glu [37]
Pomegranate	NO (at 31 μg/mL)	11.33 ± 2.3 μg/mL	Dp3,5 diglu, Cy3,5 diglu, Dp3glu [38]
Hibiscus	NO (at 218 μg/mL)	NO (at 218 μg/mL)	Dp3sam, Cy3sam [39]
Mahaleb cherry (purified) *	ND	5% (at 34 μg/mL)	Cy3glu [40]
Mahaleb cherry (crude)	ND	21% (at 39 μg/mL)	Cy3glu [40]
Black carrot (purfied) *	ND	NO (at 57.7 μg/mL)	Cy3glc [40]
Black carrot (crude)	ND	NO (at 48 μg/mL)	Cy3glc [40]
Cyanidin	7%	141 ± 1.6 μg/mL (491 μM)	
Cyanidin-3-O-galactopyranoside	ND	414 ± 2.6 μg/mL (845 μM)	

Data are expressed as mean with SEM of three replicates. Results of % inhibition are calculated to total anthocyanin content. Abbreviations are as follows: Dp3rut, delphinidin-3-O-rutinoside; Cy3rut, cyanidin-3-O-rutinoside; Dp3glu, delphinidin-3-O-glucoside; Cy3glu, cyanidin-3-O-glucoside; Dp3glc, delphinidin-3-O-galactoside; Mv3glu, malvidin-3-O-glucoside; Mv3glc, malvidin-3-O-galactoside; Dp3,5 diglu, delphinidin 3,5 diglucoside; Cy3,5 diglu, cyanidin 3,5 diglucoside; Dp3sam, delphindin-3-O-sambubioside; Cy3sam, cyanidin-3-O-sambubioside; Cy3glc, cyanidin-3-O-galactoside. ND, not determined by DNS assay; NO, no inhibition was found. * purified sample containing > 95% anthocyanins.

**Table 2:** Composition of fruit concentrates.

Type of Concentrate	Polyphenols (mg/mL)	Anthocyanins (mg/mL)	Total Sugars (mg/mL)
Blueberry	18.96 ± 0.37 ^b^	11 ± 0.08 ^a^	576.6 ± 1.7 ^b^
Cherry	9.83 ± 0.36 ^c^	3.01 ± 0.02 ^b^	502.3 ± 0.85 ^c^
Hibiscus	7.66 ± 0.15 ^c^	4.4 ± 0.03 ^b^	17.42 ± 0.74 ^d^
Pomegranate	23.32 ± 1.55 ^a^	1.85 ± 0.01 ^c^	636.5 ± 8.82 ^a^

Values are expressed as mean ± SEM. Values in the same column followed by different superscript letters are significantly different from each other (*p* < 0.05).

## Data Availability

Data are contained within the article.
